# Multi-omics investigation of perineural invasion in head and neck squamous cell carcinoma: neuroimmune mechanisms and clinical implications

**DOI:** 10.3389/fimmu.2026.1792836

**Published:** 2026-05-12

**Authors:** Muling Deng, Yuhao Lin, Chenyu Fang, Jiaqi Cai, Chuanben Chen, Jianming Ding

**Affiliations:** 1 Department of Radiation Oncology, Clinical Oncology School of Fujian Medical University, Fujian Cancer Hospital, Fuzhou, Fujian, China; 2 Department of Radiation Oncology, Clinical Oncology School of Fujian Medical University, Fujian Cancer Hospital, National Health Commission (NHC) Key Laboratory of Cancer and Metabolism, Fuzhou, China

**Keywords:** head and neck squamous cell carcinoma, immune microenvironment, multi-omics analysis, perineural invasion, super-enhancers

## Abstract

**Background:**

Perineural invasion (PNI) is an aggressive feature in head and neck squamous cell carcinoma (HNSCC), but its molecular basis and neuroimmune implications, including potential links to immunotherapy response, remain unclear.

**Methods:**

We performed an integrative multi-omics analysis using public datasets and an independent clinical cohort. Transcriptomic, proteomic, single-cell, and spatial transcriptomic data were jointly analyzed to identify PNI-associated molecular patterns, construct prognostic signatures, and characterize immune infiltration and cell-cell communication. Regulatory elements were further explored by super-enhancer mapping and target-gene prediction. Pharmacologic inhibition experiments using the TLR2 inhibitor C29 were performed in FaDu cells for functional validation.

**Results:**

PNI was associated with extracellular matrix and neuroactive signaling changes. A protein-based signature (ADIPOQ, MB, PLIN1, ADH1B) stratified survival risk. PNI-positive/high-risk tumors showed an immune-suppressed phenotype with lower predicted immunotherapy sensitivity and reduced CD8+ T-cell, B-cell, and Tfh-cell infiltration. Spatial analysis showed higher PNI scores at the invasive front, positive correlations with neural programs, and enrichment of TLR2-related signaling. TLR2 expression was associated with the PNI score, neural markers, and immune exclusion. In FaDu cells, C29 suppressed proliferation, migration, and invasion. Super-enhancer analysis identified candidate SE-target genes, including MYL4, CMYA5, and TNNT3, linked to PNI-associated biology.

**Conclusions:**

PNI in HNSCC is associated with coordinated extracellular-matrix, neuroimmune, and immune-suppressive remodeling. These findings support PNI-related molecular signatures for risk stratification and identify TLR2-related signaling and SE-associated programs as candidate mechanisms for further study.

## 1 Introduction

Head and neck squamous cell carcinoma (HNSCC) imposes a substantial global disease burden, and improvements in overall survival remain limited due to challenges such as recurrence, metastasis, and second primary malignancies, underscoring a persistent clinical need for more precise risk stratification and interpretable molecular biomarkers (1). Meanwhile, HNSCC is highly heterogeneous with respect to anatomic subsites, etiologic exposures (e.g., tobacco and alcohol) (2), and HPV-associated subtypes; for instance, HPV-related and HPV-unrelated tumors exhibit systematic differences in biological behavior and clinical outcomes (3), yet current therapeutic decision-making still relies largely on conventional clinical and histopathologic features, resulting in recurrence in a considerable proportion of patients (4). Therefore, investigations centered on key invasive phenotypes, incorporating mechanistic and translational perspectives, represent an important avenue to advance prognostic assessment and optimize treatment strategies.

Among these invasive phenotypes, perineural invasion (PNI) is regarded as a critical route of tumor dissemination. Conceptually, PNI refers to the invasion of perineural structures by tumor cells with subsequent extension along nerve-associated spaces and/or nerve sheath compartments (5, 6); multidisciplinary reviews have emphasized the need for a clear and standardized definition to minimize inconsistencies in terminology across disciplines (5). In head and neck malignancies, PNI is relatively prevalent and has been associated with poorer survival outcomes as well as challenges in achieving complete tumor eradication. Accumulating evidence further suggests that PNI is not merely a “passive conduit,” but rather reflects an invasive niche co-shaped by tumor–nerve interactions (cancer-nerve crosstalk) and the local microenvironment (7). In cohorts such as oral squamous cell carcinoma and oral tongue squamous cell carcinoma, multiple systematic reviews and meta-analyses have further supported associations between PNI and an increased risk of locoregional recurrence, along with worse outcomes including overall survival (OS) and disease-free survival (DFS), thereby reinforcing its clinical relevance as a high-risk histopathologic feature (8).

Although the clinical significance of perineural invasion (PNI) has been repeatedly demonstrated, substantial gaps remain in its detection and management. On the one hand, PNI is most often confirmed on postoperative histopathologic examination, which limits reliable pre-treatment risk stratification and constrains the development of mechanism-informed, targeted interventions. On the other hand, radiologic assessment related to “perineural spread” also presents challenges—for example, a classic study reported that MRI is highly sensitive for detecting the presence of perineural spread, yet may be insufficient for delineating its full extent, suggesting that subtle or microscopic invasion can still be underestimated (9). More importantly, despite growing recognition of the importance of PNI, existing molecular evidence remains relatively fragmented, and robust biomarkers that can directly inform clinical stratification and intervention are still lacking.

This study aims to elucidate the molecular mechanisms underlying PNI in HNSCC and to provide insights to improve prognostic assessment and inform the development of targeted therapeutic strategies for PNI.

## 2 Methods

### 2.1 Data collection

This study utilized data from multiple publicly available databases. The TCGA-HNSC dataset was obtained from the TCGA database and includes 566 HNSCC samples, covering gene expression and clinical information. Clinicopathological variables, including HPV status, pathologic stage, T stage, N stage, grade, age, and sex, were extracted when available and summarized in **Supplementary Table S1**. Immune phenotype scoring data were sourced from the TCIA database, which provides immune genomic analysis results for 20 types of solid tumors in TCGA. Tumor proteomic data were acquired through the PDC platform (https://pdc.cancer.gov/pdc/), focusing on the proteomics of HNSCC. The single-cell dataset GSE195832 was obtained from the GEO database (https://www.ncbi.nlm.nih.gov/geo/), including 8 samples (pre- and post-treatment with nivolumab). The spatial transcriptomic dataset was obtained were obtained from GSE181300, including 4 sections from the primary locations and 4 from the invasive front regions of primary sites.

In our center, data for 85 patients with both an available PNI status and survival results were used to analyze the effects of PNI on prognosis. This retrospective study included 85 patients with head and neck squamous cell carcinoma (HNSCC) who underwent surgical treatment at Fujian Cancer Hospital between July 2013 and December 2021. Eligible patients met the following criteria: (1) pathologically confirmed HNSCC; (2) radical resection of the primary lesion with a complete postoperative pathology report; (3) available pathological assessment of perineural invasion (PNI) status; and (4) complete clinicopathological and follow-up data. (5) patients with locally advanced head and neck squamous cell carcinoma (HNSCC) who underwent curative-intent surgery followed by risk-adapted postoperative radiotherapy (PORT) or postoperative concurrent chemoradiotherapy (POCRT) were included. Patients were excluded if they had (1) non-squamous histology; (2) recurrent disease, a second primary tumor, or synchronous malignancies; (3) biopsy only, palliative surgery, or inadequate pathological evaluation; (4) missing key clinicopathological variables, PNI status, or follow-up data. This study was approved by the Ethics Committee of Fujian Cancer Hospital (K2024-301-01).

### 2.2 Differential expression analysis

Differential analyses were performed on RNA-seq and proteomics data to identify differentially expressed genes (DEGs) and differentially abundant proteins (DEPs), respectively. Gene expression profiles and clinical annotations for the TCGA-HNSC cohort (n = 566) were obtained as described in Data Collection. Samples were grouped by PNI status. Differential expression between PNI-positive and PNI-negative tumors was performed with DESeq2 in R (v4.1.2) following the standard workflow. The p-value was adjusted by the Benjamini–Hochberg method to control for the false discovery Rate (FDR). Genes with the cut-off criteria of |logFC| ≥ 1.0 and adj. P < 0.05 were regarded as DEGs. Protein intensities were log2-transformed and differential abundance tested with limma (v3.50.0). The p-value was adjusted by the Benjamini–Hochberg method to control for the false discovery Rate (FDR). Proteins with the cut-off criteria of |logFC| ≥ 1.0 and adj. P < 0.05 were regarded as DEPs.

### 2.3 Enrichment analysis

Gene Ontology (GO), Kyoto Encyclopedia of Genes and Genomes (KEGG), and Gene Set Enrichment Analysis (GSEA) were performed for functional enrichment analysis. All analyses were conducted using the R package “clusterProfiler”.

### 2.4 Construction of PPI

In our study, we used the Search Tool for the Retrieval of Interacting Genes online tool (STRING), which is designed for predicting protein–protein interactions (PPI), to construct a PPI network of selected genes. Interactions were retained at a minimum combined score of 0.40. Proteins connected by edges meeting this threshold were considered network members, and those passing this interaction criterion were treated as neuro-immune-related key nodes.

### 2.5 Construction and validation of prognostic markers

Univariate Cox regression analysis was first performed to screen genes associated with prognosis, and genes with P < 0.05 were selected as candidate prognostic genes. Because transcriptomic/proteomic data are high-dimensional and direct multivariable Cox regression may lead to substantial overfitting, LASSO regression (R package glmnet) was first applied to reduce redundant variables through L1 regularization. The optimal penalty parameter λ was selected using the 1-standard error (1-SE) rule. Since LASSO is based on a linear framework and may not fully capture nonlinear relationships or feature interactions in biological systems, a random forest model was additionally used to rank feature importance and identify variables with robust predictive value. Features retained by LASSO and prioritized by random forest were subsequently entered into a multivariable Cox regression model to construct the final prognostic signature. The risk score for each sample was calculated based on the regression coefficients derived from the multivariable Cox model.

### 2.6 Association of PNI with clinicopathological variables and survival

Associations between PNI and clinicopathological variables were evaluated in the institutional cohort. Univariate analyses were first performed to examine the relationships between PNI and available clinicopathological features, including T stage, N stage, grade, HPV/p16 status, smoking history, margin status, extranodal extension (ENE), lymphovascular invasion (LVI), AJCC stage, and primary tumor site, where available. Variables of clinical relevance were then entered into a multivariable logistic regression model to identify factors independently associated with PNI. Forest plots were used to visualize odds ratios and 95% confidence intervals. In addition, supplementary analyses were performed to evaluate the mechanistic relevance of the protein-based risk signature to PNI, including associations between risk score and PNI status, PNI-related signatures, and nerve-related gene signatures.

### 2.7 Immune microenvironment analysis

The proportion of immune cell infiltration was calculated using CIBERSORT. Principal component analysis (PCA) was used to analyze the distinguishability of immune cell infiltration between disease and control groups, and Spearman correlation analysis was conducted to assess the relationships between immune cells. CIBERSORT analysis was performed using the R package IOBR, and visualized with ggplot2. The correlation between immune cell infiltration levels was computed using the R package psych and visualized with the R package corrplot. Additionally, PCA was performed using the R package FactoMineR and visualized with factoextra. For the comparisons of immune cell infiltration, statistical significance was assessed using Student’s t test.

### 2.8 Single-cell analysis

Single-cell data analysis was performed using the R package Seurat. To remove low-quality cells and low-expressed genes, the following thresholds were set: the number of features per cell was between 200 and 3000, the count range was between 500 and 2000, and mitochondrial gene proportion was less than 10%. Data normalization was performed using the NormalizeData function, and 3000 highly variable genes were identified using the vst method in the FindVariableFeatures function. Batch effects were corrected using the R package harmony. The effectiveness of batch correction was evaluated by comparing UMAP embeddings before and after Harmony correction, with particular attention to the sample/batch distribution of cells (**Supplementary Figures S1**, **S2**).

After dimensionality reduction by PCA, the top 50 principal components were selected for subsequent analysis. We constructed a Shared Nearest Neighbor (SNN) graph and performed cell clustering using FindClusters (resolution set to 0.7). Cell clusters were annotated as known cell types utilizing canonical marker genes. The marker genes used for cell type annotation are listed in **Supplementary Table S2**. Visualization was carried out using RunUMAP. Feature genes were identified using the FindAllMarkers function, and top feature genes were displayed using a bubble plot. The proportion differences between different cell types were compared, and statistical significance was evaluated by chi-squared test.

Cell communication was inferred using the R package CellChat. The CellChat object was constructed using the createCellChat function, high-expressed genes were identified using the identify over Expressed Genes function, and over-expressed ligand-receptor interaction pairs were identified using the identify over Expressed Interactions function. Cell communication probabilities between subpopulations were computed using the computeCommunProb function and visualized using netVisual_bubble and other functions. To compare communication differences between prognostic groups, cells were stratified into high-risk and low-risk groups according to the risk score derived from the prognostic model.

To further investigate the functional relevance of risk-associated neuroimmune genes, in silico knockout analysis was performed using scTenifoldKnk. Based on the prognostic signature, ADH1B, ADIPOQ, MB, and PLIN1 were selected as neuroimmune genes for perturbation analysis, and transcriptomic changes after simulated knockout were subjected to downstream functional enrichment analysis.

### 2.9 Spatial transcriptomic data download and preprocessing

Spatial transcriptomic data quality control and alignment were performed using spaceranger-2.1.1. Processed spatial transcriptomic sample data (gene-spot matrix) were analyzed using Seurat. SCTransform was used to standardize the raw count data of different spots. Spatial transcriptomic data were deconvolved using Robust Cell Type Deconvolution (RCTD), and cell types were assigned to spatial spots using the doublet model.

A PNI-associated signature score (“PNI score”) was calculated based on the expression of ADH1B, ADIPOQ, MB, and PLIN1, which were derived from the prognostic model and used here as a PNI-related molecular signature. This score was used to quantify the relative activity of the PNI-associated transcriptional program across spatial regions and should be interpreted as a molecular signature score rather than a direct histopathological measurement of perineural invasion. Correlation analyses were further performed between the PNI score and predefined neural gene modules, as well as between TLR2 expression and the PNI score and related module scores across spatial sections.

Cell-cell communication analysis in spatial transcriptomic data was performed using CellNEST with default parameters, including threshold_gene_exp = 98 and filter_min_cell = 1. Statistical significance was defined as P < 0.05. Differential pathways identified by CellNEST were ranked by significance.

### 2.10 Super enhancer analysis

Super enhancers (SEs) were defined based on HNSCC ChIP-seq profiles of H3K4me1 and H3K27ac, two canonical enhancer-associated histone marks. Publicly available datasets were obtained from GEO (GSE149043 and GSE103554). Epigenomic peaks were called using MACS2, and overlapping peak intervals across samples/marks were merged and treated as the same peak region. SEs were subsequently identified using the ROSE algorithm based on stitched enhancer regions and signal ranking. The ROSE analysis was performed using default parameters, including a stitching distance of 12,500 bp and a transcription start site exclusion window of 0 bp.

To investigate three-dimensional chromatin organization, Hi-C data were analyzed using HiCExplorer, and topologically associating domains (TADs) were identified at 25-kb resolution.Genome-wide Hi-C data were processed using Juicer to support downstream 3D genome interpretation of enhancer–target relationships. Differentially expressed eRNAs were defined using a threshold of P < 0.05 and log2 fold change > 0.75. Correlation analyses were also performed between predicted SE-target gene expression and the PNI score and related pathway module scores.

### 2.11 EdU assay

The EdU Cell Proliferation Kit (Beyotime, China) was used to assess cell proliferation. Briefly, FaDu cells treated with DMSO or the TLR2 inhibitor C29(#HY-100461, MCE) were seeded into 24-well plates and incubated with EdU reagent. Cells were then fixed with 4% paraformaldehyde (Sigma, USA), permeabilized with 0.5% Triton X-100, and counterstained with DAPI. Images were captured using an inverted fluorescence microscope (ZEISS, Germany), and EdU-positive cells were counted.

### 2.12 Colony formation assay

FaDu cells treated with DMSO or C29 were seeded into 6-well plates and cultured for approximately 14 days. The colonies were then fixed with 4% paraformaldehyde (Sigma, USA), stained with crystal violet (Beyotime, China), photographed, and counted.

### 2.13 Wound-healing assay

FaDu cells treated with DMSO or C29 were seeded into 6-well plates, and a linear wound was generated using a pipette tip. Wound closure was photographed at 0 and 24 h to assess cell migration.

### 2.14 Transwell invasion assay

Transwell chambers (Corning Incorporated, Life Sciences, USA) coated with Matrigel (BD, USA) were used for the cell invasion assay. Briefly, FaDu cells treated with DMSO or C29 were added into the upper chamber. After incubation, the invaded cells were fixed with 4% paraformaldehyde (Sigma, USA), stained with crystal violet (Beyotime, China), photographed, and counted.

### 2.15 Statistical analysis

All statistical analyses were performed in R version 4.1.2. For the significance comparison of gene expression, immune cell infiltration ratios, and other data, the Wilcoxon rank-sum test was used for two-group comparisons, and the Kruskal-Wallis test was used for multiple-group comparisons. Prognostic analysis was conducted using Kaplan-Meier survival curves, and the log-rank test was used to assess the significance of survival differences. Cox proportional hazards models were used for univariate and multivariable survival analyses. Correlation analyses were performed using Spearman correlation unless otherwise specified. For plotting, p-values were represented as ns (p > 0.05), *(p < 0.05), **(p < 0.01), ***(p < 0.001), **** (p < 0.0001).

## 3 Results

### 3.1 PNI in HNSC and its association with prognosis

**Figure 1:**
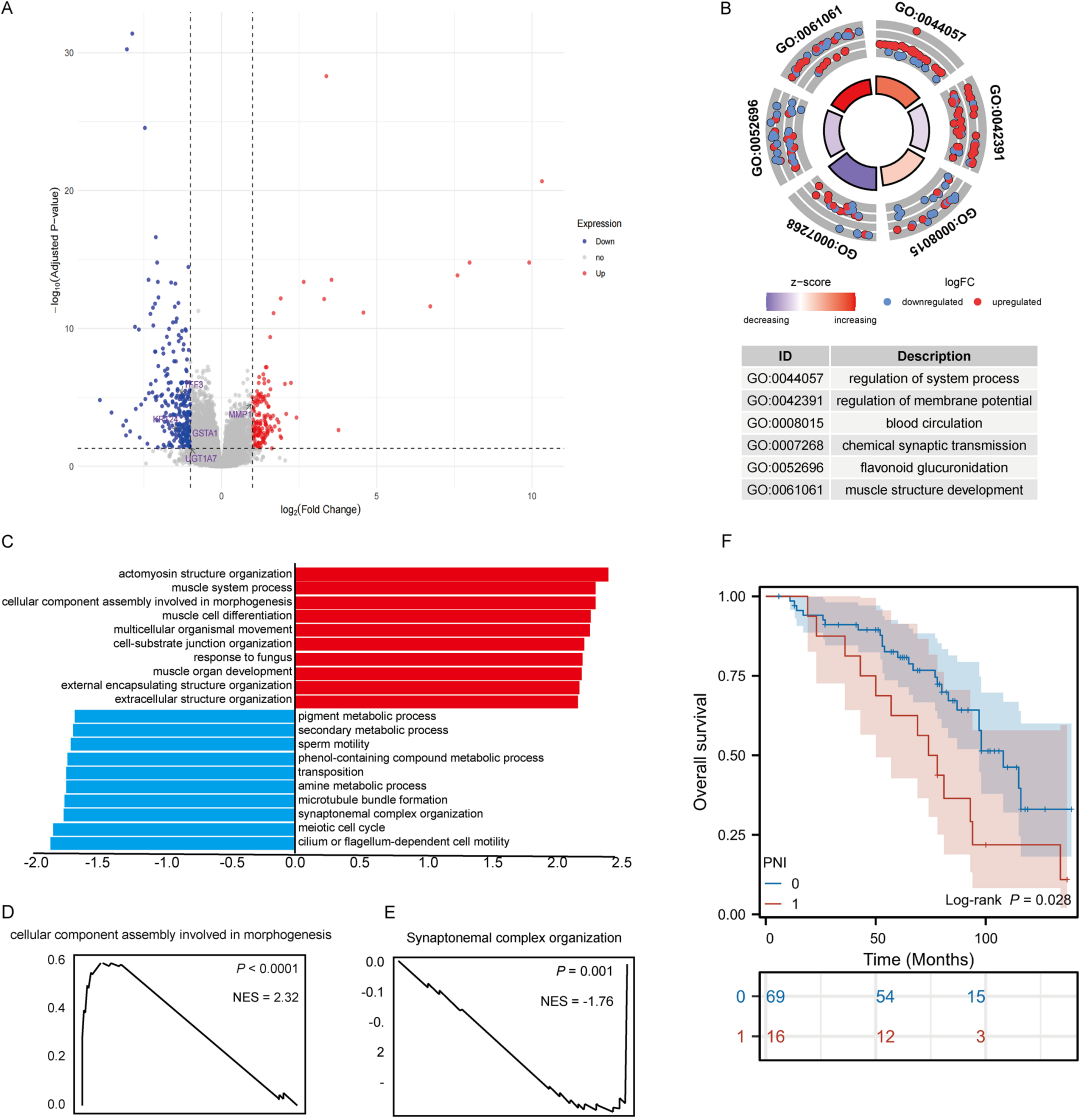
Transcriptomic characterization and clinical prognostic relevance of PNI in HNSCC. **(A)** Volcano plot of differentially expressed genes (DEGs) between PNI-positive and PNI-negative tumors (red, upregulated in PNI; blue, downregulated). **(B)** Gene Ontology (GO) enrichment visualization of PNI-associated DEGs. **(C-E)** Gene set enrichment analysis (GSEA) showing pathways enriched in PNI-positive versus PNI-negative tumors. **(F)** Kaplan–Meier overall survival curves comparing PNI-positive and PNI-negative patients in the Fujian Cancer Hospital cohort.

**Figure 2:**
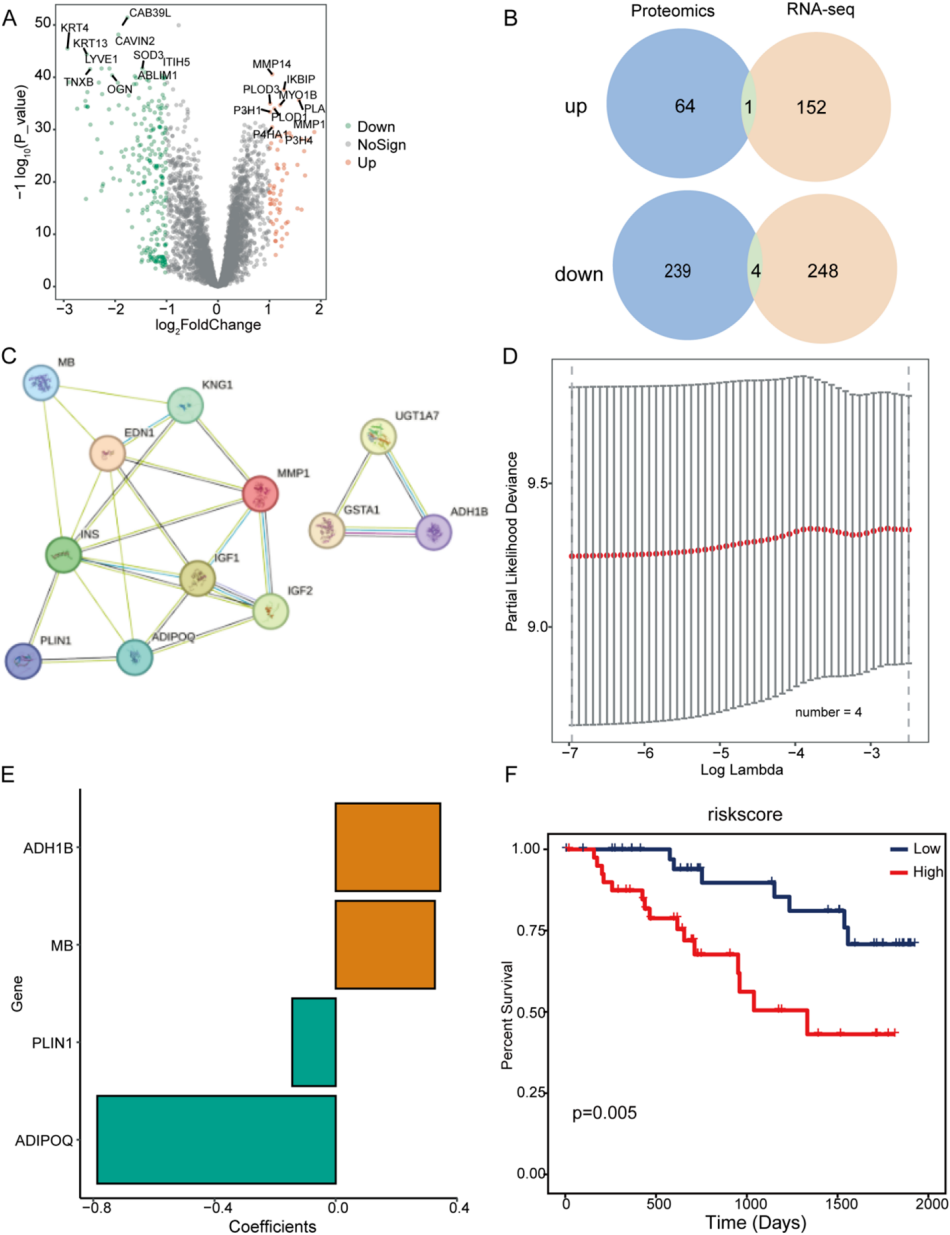
Integrated proteogenomic analysis of PNI status in HNSCC patients. **(A)** Volcano plot of differentially expressed proteins between groups. **(B)** Overlap of concordant transcriptomic and proteomic changes. **(C)** PPI network of PNI-related neuroimmune hub genes/proteins. **(D)** LASSO cross-validation for optimal λ selection. **(E)** LASSO coefficients of selected prognostic features. **(F)** Kaplan–Meier overall survival curves for high- vs. low-risk groups.

Differential expression analysis of the TCGA-HNSC cohort, grouped by PNI status, identified 405 differentially expressed genes (153 upregulated, 252 downregulated; **Figure 1A**). Enrichment analysis revealed upregulated genes were mainly associated with actin binding and cardiac muscle contraction, while downregulated genes were linked to neuroactive ligand-receptor interactions (**Figures 1B–E**; **Supplementary Figures S3**, **4**). At the proteomic level (PDC dataset), 308 differentially expressed proteins were identified (65 upregulated, 243 downregulated; **Figure 2A**). Downregulated proteins were involved in contractile fiber structures, while upregulated proteins were enriched in collagen-containing extracellular matrix pathways and related pathways (**Supplementary Figures S5**, **6**).

Cross-validation showed that MMP1 was upregulated in both transcriptomic and proteomic levels, while TFF3, KRT24, GSTA1, and UGT1A7 were downregulated in both (**Figure 2B**), suggesting these molecules may form a robust PNI-associated molecular pattern. PPI network analysis using STRING identified 9 key nodes (EDN1, IGF1, IGF2, INS, KNG1, ADIPOQ, MB, PLIN1, ADH1B; **Figure 2C**). LASSO-Cox modeling using these proteins calculated the risk score, dividing patients into high- and low-risk groups. Multivariable Cox and survival analysis showed ADIPOQ, MB, PLIN1, and ADH1B were significantly associated with overall survival (**Figures 2D–F**; **Supplementary Figure S7**). Clinical characteristic distribution based on the risk score is shown in **Supplementary Figure S8**. At our center, 69 patients were in the PNI-negative group and 16 were in the PNI-positive group. Kaplan-Meier analysis demonstrated that patients with PNI had significantly worse OS than those without PNI (log-rank P = 0.028; **Figure 1F**). To assess whether PNI simply reflected overall tumor aggressiveness, we examined its associations with clinicopathological variables. In univariate analysis, PNI was associated with several adverse pathological features (**Supplementary Figure S9**). In multivariable logistic regression, oral cavity subsite, ENE, and LVI remained independently associated with PNI, whereas T stage, N stage, and grade were not significant (**Supplementary Figure S10**). These findings suggest that PNI is not merely a surrogate for advanced tumor stage.

### 3.2 Association of PNI with immunotherapy response and immune characteristics

**Figure 3:**
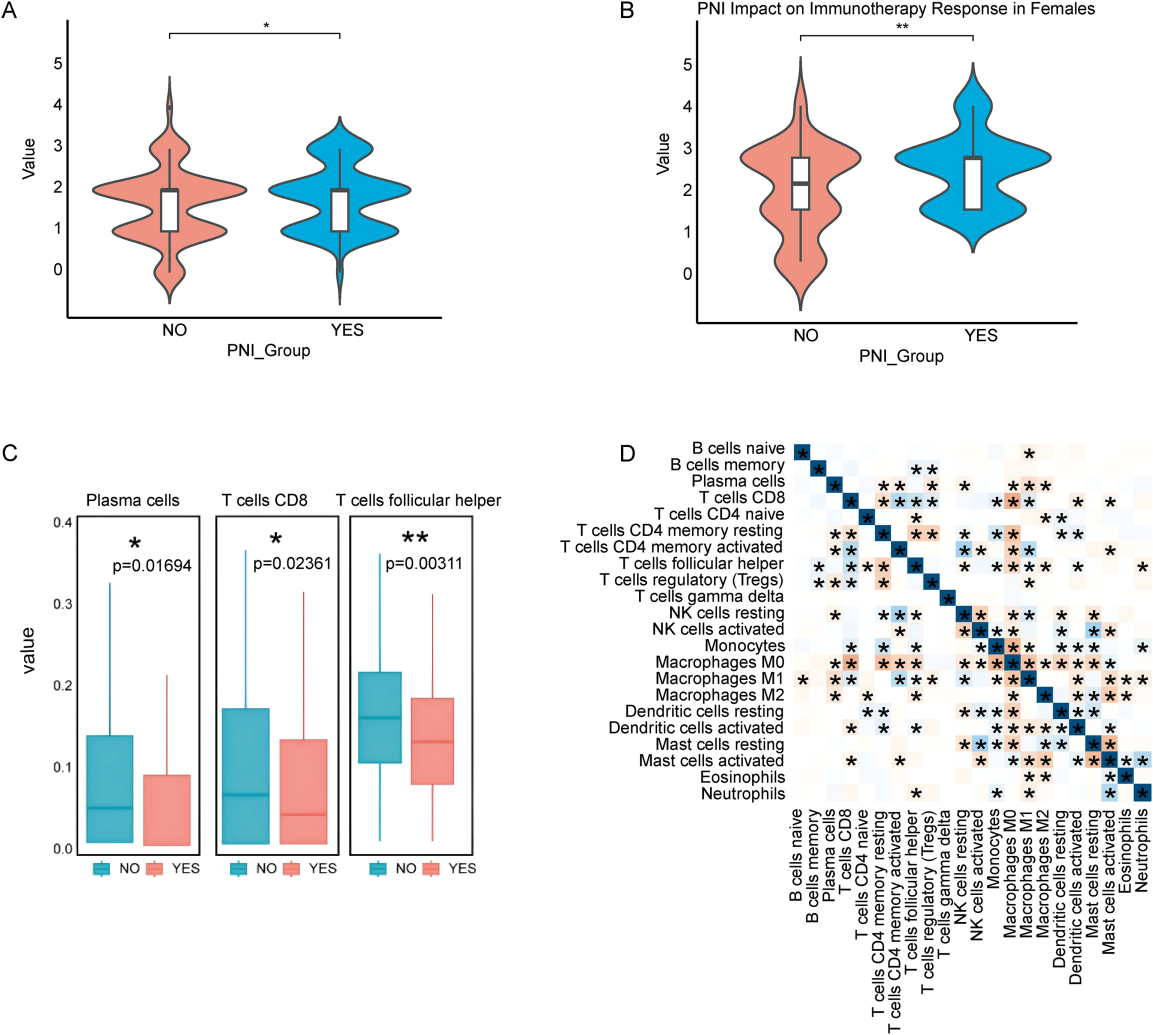
PNI associates with immunotherapy response and immune infiltration characteristics in HNSCC. **(A, B)** Sex-stratified association between PNI status and immunotherapy response/immune phenotyping profiles (male and female cohorts, respectively). **(C)** Differential infiltration of three immune cell types. **(D)** Correlation matrix of immune cell infiltration proportions across samples. The symbol "*" represents a p-value less than 0.05, and "**" represents a p-value less than 0.01, both indicating statistically significant differences.

In HNSCC, PNI status is closely related to immune therapy response. Analysis of 528 HNSCC records from the TCIA database, including immune phenotyping scores, revealed a significant difference in immune therapy response based on PNI status. PNI-positive patients exhibited lower sensitivity to immune therapy (**Figures 3A, B**).

**Figure 4:**
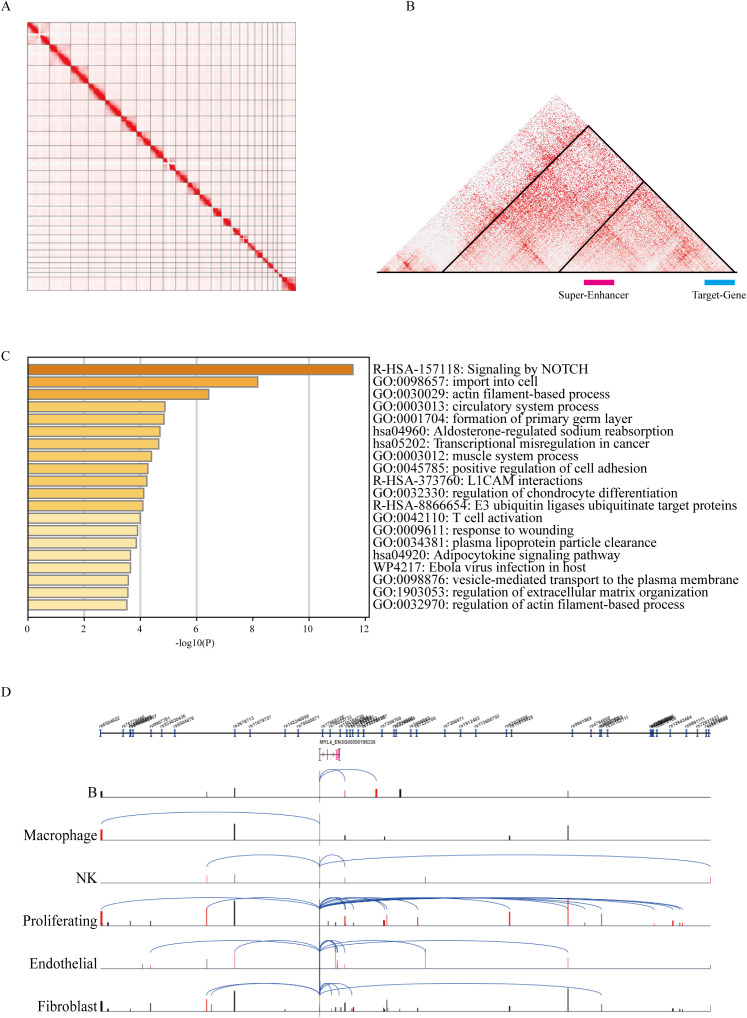
Identification of super-enhancer target genes and analyses of super-enhancer activation and chromatin architecture. **(A)** Genome-wide Hi-C contact map of HNSC tumor tissues. **(B)** Zoomed-in view of the Hi-C map, where the triangular region indicates a topologically associating domain (TAD). **(C)** Functional enrichment analysis of the predicted target genes. **(D)** Super-enhancer activation patterns across different cell types.

Further analysis of immune cell infiltration in the tumor microenvironment, using the CIBERSORT algorithm to quantify 22 immune cell types, showed that B cells, CD8+ T cells, and follicular helper T cells were significantly more infiltrated in the PNI-negative group compared to the PNI-positive group (p < 0.05) (**Figure 3C**; **Supplementary Figure S11**). Correlation analysis revealed a significant relationship between CD8+ T cell infiltration and M0 and M1 macrophages (**Figure 3D**). These results align with the activation of cell types identified by super-enhancers (**Figure 4D**), suggesting that CD8+ T cells may interact with neuroimmune processes in PNI and influence immune therapy response and clinical prognosis.

**Figure 5:**
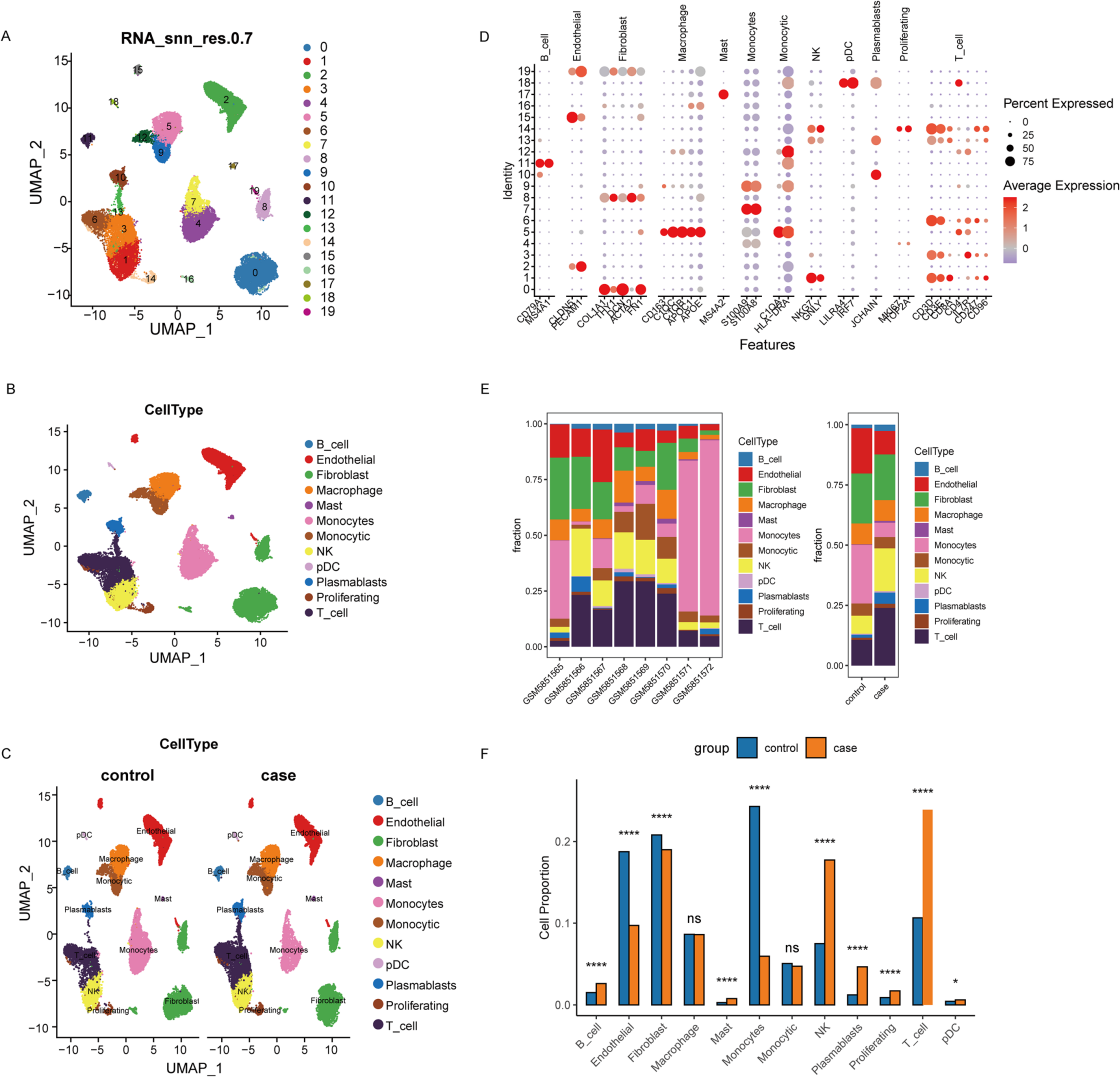
Single-cell transcriptomic landscape and cell-type composition changes. **(A)** UMAP embedding of all single cells colored by clusters. **(B)** UMAP embedding colored by sample origin. **(C)** UMAP embedding colored by experimental group. **(D)** Bubble plot of canonical marker genes used for cell-type annotation. **(E)** Overall cell-type proportion across samples/groups. **(F)** Comparison of cell-type proportions between pre-treatment and post-treatment samples. The symbol "*" represents a p-value less than 0.05, and "****" represents a p-value less than 0.0001, indicating statistical significance and very strong statistical significance, respectively.

**Figure 6:**
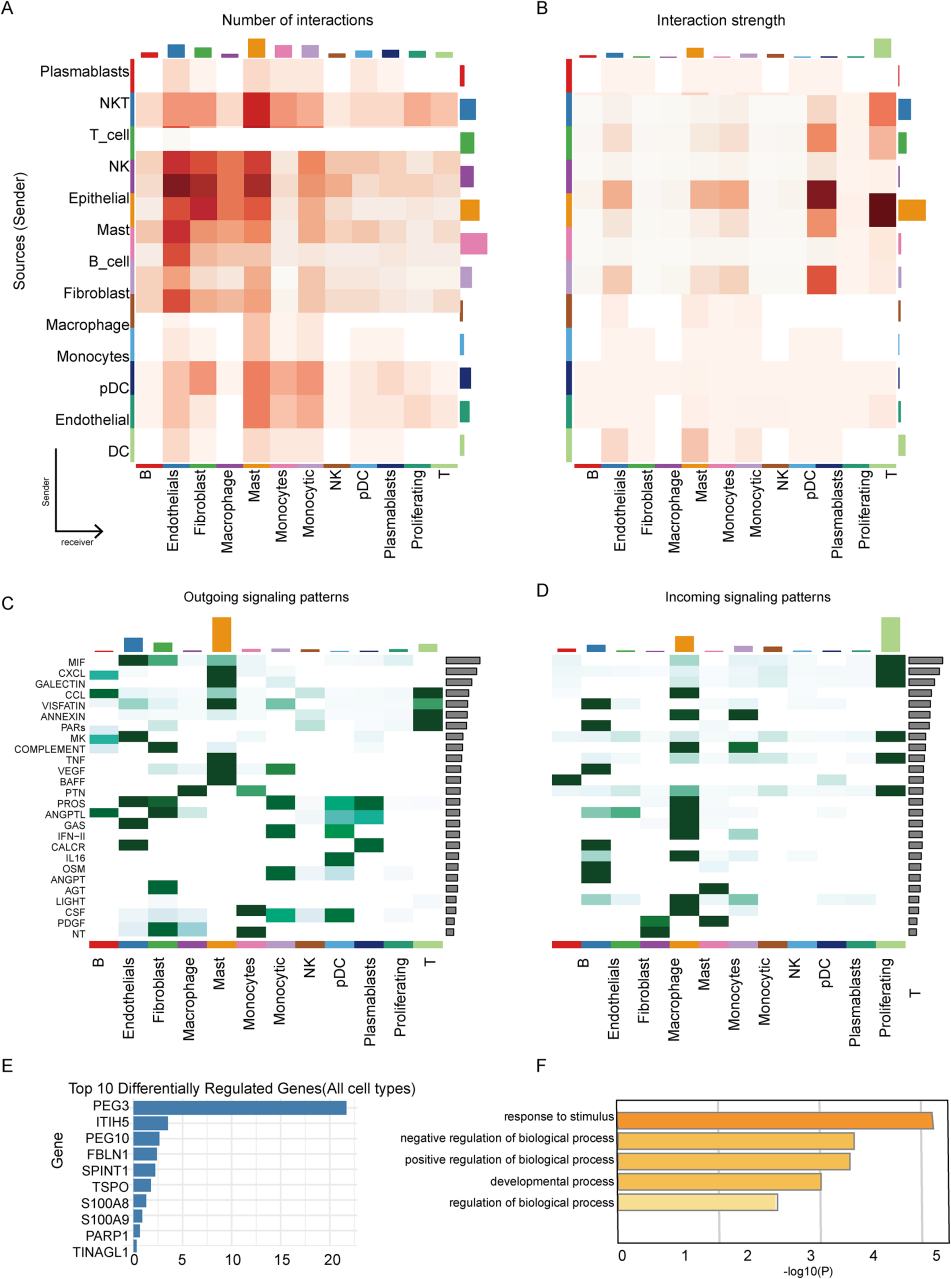
Cell–cell communication analysis reveals the impact of neuroimmune genes on patients. **(A, B)** Altered intercellular interaction strength between major cell populations (red, increased in low-risk; blue, decreased). **(C, D)** Differential signaling pathways in cell–cell communication. **(E)** Top 10 genes most affected by in silico knockout of neuroimmune genes (scTenifoldKnk). **(F)** GO clustering results of the top affected genes shown in **(E)**.

Single-cell transcriptomic analysis identified 19 major cell clusters (**Figure 5A**). UMAP visualization showed the distribution of these clusters across different samples (**Figure 5B**) and within major cell types (**Figure 5C**). Using classical marker genes, we annotated these clusters as primary immune and stromal cell populations, including fibroblasts, monocytes, NK cells, and T cells (**Figure 5D**). Comparing the cell composition before and after nivolumab treatment revealed significant changes in the proportions of fibroblasts, monocytes, NK cells, and several T cell subsets (**Figures 5E, F**). Based on the previously established multivariable Cox model, cells were categorized into high-risk and low-risk groups. Cell-cell communication analysis showed a marked reduction in interactions among macrophages, fibroblasts, and endothelial cells. (**Figures 6A, B**). In contrast, T cells in the low-risk group showed significantly enhanced MIF and CXCL signaling pathways (**Figures 6C, D**), suggesting that increased immune interactions may correlate with better clinical outcomes.

To further explore the functional roles of PNI genes, we performed simulated gene knockout analysis using the scTenifoldKnk tool on the single-cell data. The results revealed widespread transcriptomic changes due to the disturbance of neuroimmune genes (**Figure 6E**), with GO enrichment analysis indicating that these genes are primarily involved in neurogenesis and developmental regulation (**Figure 6F**).

### 3.3 Spatial transcriptomics and pharmacologic validation identify TLR2 signaling as a driver of PNI-associated progression

**Figure 7:**
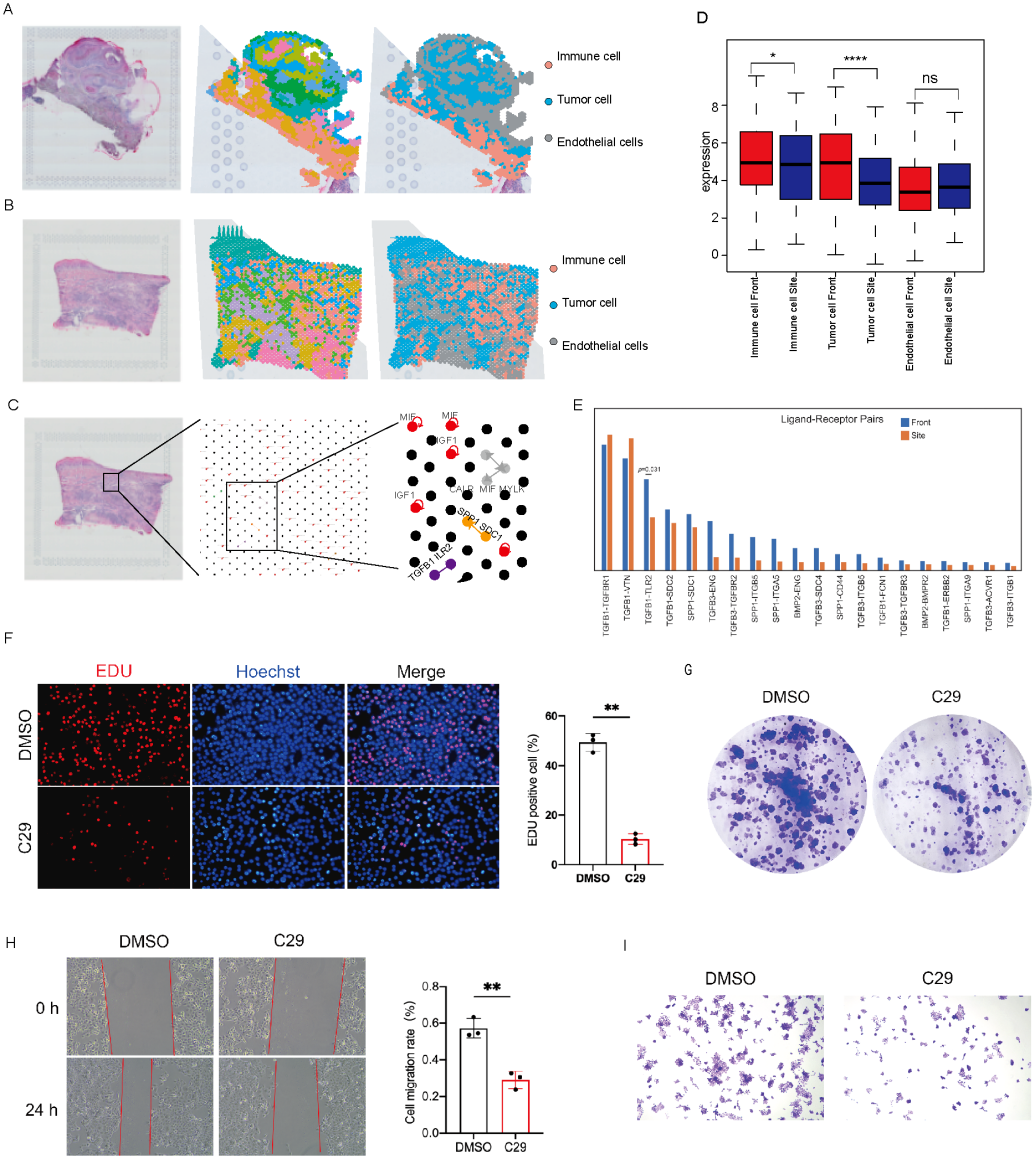
Spatial transcriptomic analysis and pharmacologic validation support the association of TLR2-related signaling with PNI-associated progression. **(A, B)** Spatial transcriptomic cell population distribution. **(C)** Cell-cell communication dot plot inferred by CellNEST. **(D)** Comparison of PNI scores between tumor core and invasive front. **(E)** Differential communication signals/pathways between tumor core and invasive front. **(F)** EdU staining and quantification showing reduced proliferation in FaDu cells after C29 treatment. **(G)** Colony formation assay showing impaired clonogenic growth after C29 treatment. **(H)** Wound-healing assay and quantification showing decreased migratory ability after C29 treatment. **(I)** Transwell invasion assay showing reduced invasive ability of FaDu cells after C29 treatment. The symbol "*" represents a p-value less than 0.05, "**" represents a p-value less than 0.01, and "****" represents a p-value less than 0.0001, all indicating different levels of statistically significant differences.

By integrating and analyzing spatial transcriptomic data (GSE181300), we identified three major cell populations in the tumor microenvironment (**Figures 7A, B**). A comparison of PNI scores between the tumor core and invasive front showed a significant increase in PNI levels at the invasive front (**Figure 7D**), suggesting stronger interactions between nerves and tumors at sites of local progression. The PNI score also showed positive correlations with predefined neural gene modules across multiple sections, supporting its relevance to PNI-associated neural programs (**Supplementary Figure S12**). To further assess the relevance of TLR2-related signaling, we correlated TLR2 expression with the PNI score and related module scores. TLR2 expression was positively correlated with the PNI score and neural marker scores, particularly in invasive-front sections, and negatively correlated with immune exclusion in Front1 and Front2 (**Supplementary Figure S13**). These findings support a spatial association between TLR2-related signaling and PNI-associated neuroimmune features, rather than a direct causal role.

To explore the molecular mechanisms, we performed cell-cell communication analysis on spatial samples using the CellNEST attention network framework (**Figure 7C**). Differential communication analysis revealed distinct communication patterns between the tumor core and the invasive front (**Figure 7E**). Notably, TLR2-related signaling pathways were significantly enriched at the invasive front, supporting a potential association of TLR2-related signaling with local tumor-neuron interactions and progression.

To further validate the functional involvement of TLR2 signaling suggested by the spatial transcriptomic analysis, we performed pharmacologic inhibition experiments in FaDu cells using the TLR2 inhibitor C29. EdU assays showed that C29 markedly reduced DNA synthesis, as evidenced by a significant decrease in the proportion of EdU-positive cells compared with the DMSO control (**Figure 7F**). Consistently, colony formation assays demonstrated that C29 impaired clonogenic growth, with fewer and smaller colonies observed after C29 treatment (**Figure 7G**). Moreover, wound-healing assays revealed that C29 significantly suppressed the migratory capacity of FaDu cells, as indicated by delayed wound closure at 24 h (**Figure 7H**). Transwell invasion assays further showed that C29 treatment significantly reduced the number of invading cells, supporting an inhibitory effect on the invasive phenotype of FaDu cells (**Figure 7I**). Collectively, these findings provide experimental support for the involvement of TLR2 signaling in promoting the proliferative and invasive properties of HNSCC cells and are consistent with our spatial transcriptomic results showing enrichment of TLR2-related signaling at the invasive front.

### 3.4 Expression of PNI-associated enhancers across different HNSCC cell types

**Figure 8:**
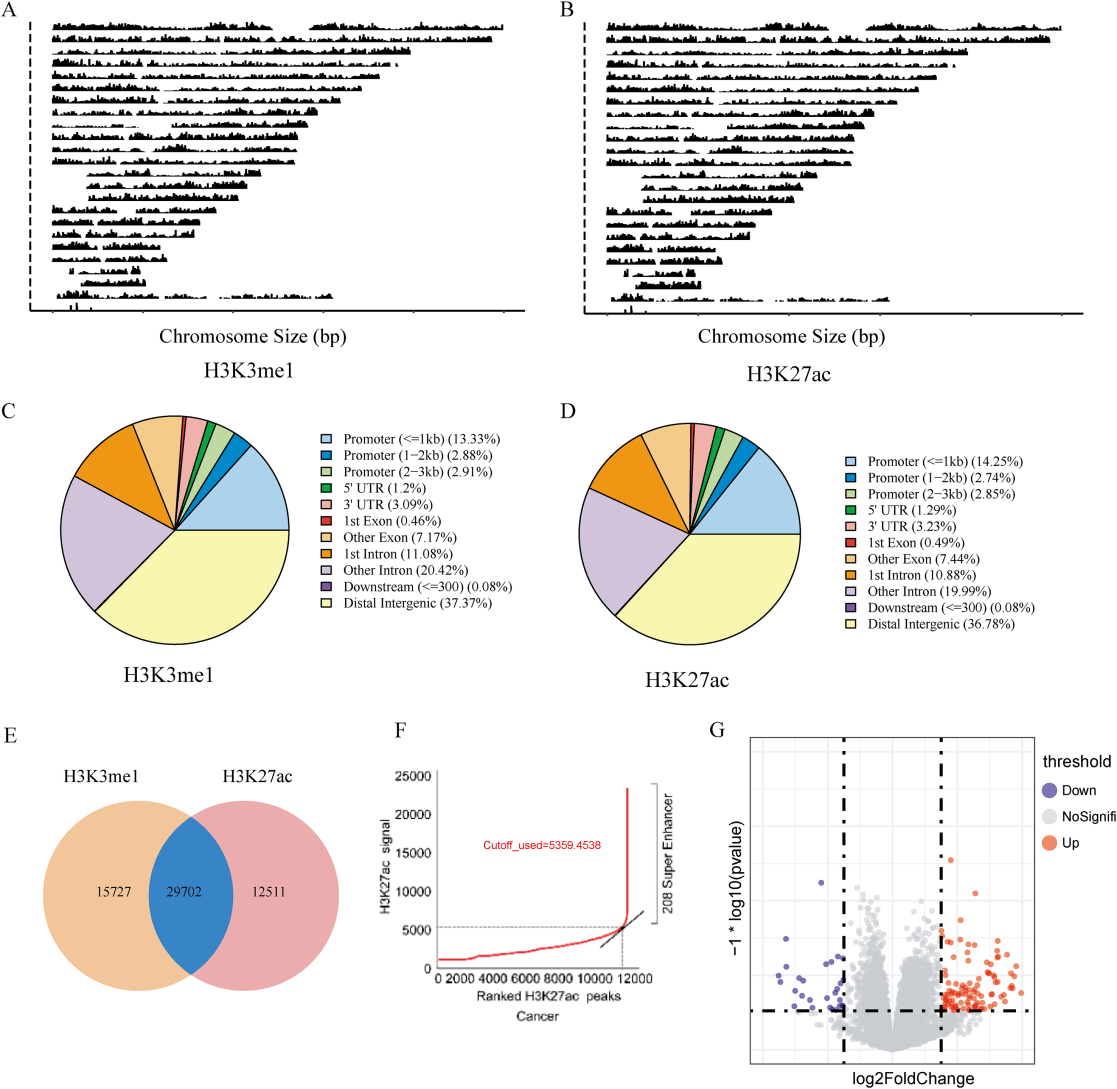
Epigenomic identification of active super-enhancers in HNSCC. **(A, B)** Genome-wide peak distribution of H3K4me1 and H3K27ac, respectively. **(C, D)** Genomic location distribution of H3K4me1 and H3K27ac peaks. **(E)** Overlap of candidate enhancer loci identified by both histone marks. **(F)** Super-enhancers identified by the ROSE algorithm. **(G)** Volcano plot showing differential expression of super-enhancer eRNAs between tumor and normal tissues.

To explore the molecular mechanisms of HNSCC, we first used ChIP-seq data for H3K4me1 and H3K27ac to identify potential super-enhancer locations across the genome. By analyzing peak identification and position distribution (**Figures 8A–D**), we identified regions within ±2Mb of gene promoters with more than 5 peaks as candidates for further analysis. Using this criterion, H3K4me1 data identified 45,429 sites, and H3K27ac data identified 42,213 sites. After overlapping the data, 29,702 sites were identified, including 5,712 potential super-enhancer regions (**Figure 8E**). ROSE(Regions of Overlap with Super Enhancers) algorithm analysis identified 208 potential super-enhancers (**Figure 8F**). By integrating eRNA expression data with previously obtained enhancer location information, we successfully identified 123 active super-enhancers in HNSCC, providing important targets for further research into the epigenetic regulatory mechanisms of HNSCC. (**Figure 8G**).

Enhancers generally increase gene expression by bringing target genes closer through DNA 3D structure (Loop formation) (10, 11). Therefore, analyzing the TAD structure in Hi-C data for HNSC helps identify target genes of super-enhancers. Further Hi-C analysis predicted 131 super-enhancer-target gene pairs (**Figures 4A, B**). Gene clustering analysis revealed that these genes are associated with T cell activation, ubiquitination, and neurodevelopment (**Figure 4C**).

**Figure 9:**
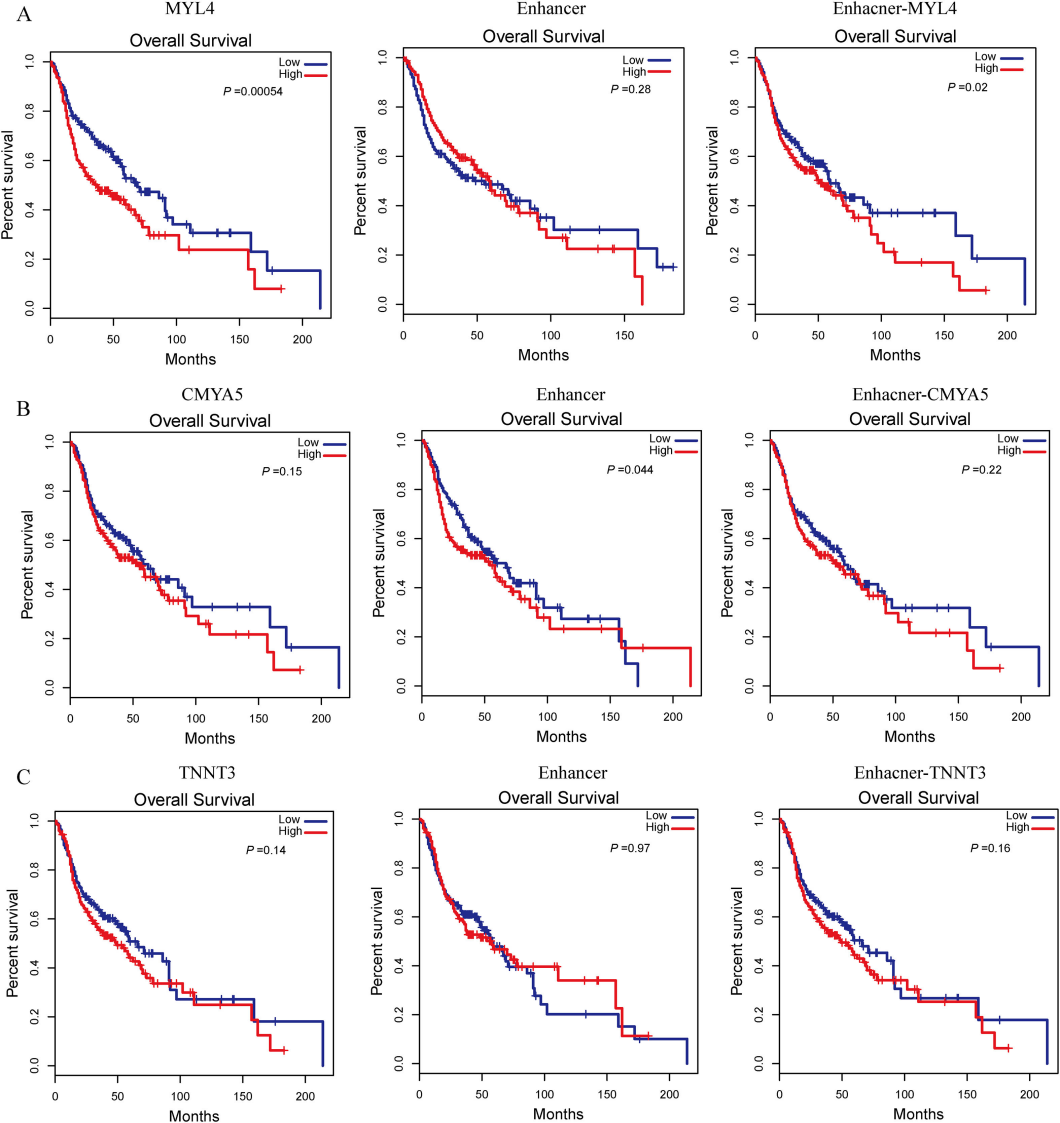
Survival analyses of three super-enhancer–target gene pairs in HNSC patients. **(A)** Overall survival analysis of the MYL4 super-enhancer–target gene pair. **(B)** Overall survival analysis of the CMYA5 super-enhancer–target gene pair. **(C)** Overall survival analysis of the TNNT3 super-enhancer–target gene pair.

Among the PNI-related genes, we identified 3 super-enhancer-target gene pairs (chr17:45390982-MYL4, chr5:79065952-CMYA5, chr11:1861215-TNNT3). Survival analysis showed that chr17:45390982-MYL4 significantly affected patient survival (**Figures 9A–C**). To further assess the potential relevance of these predicted SE-target genes to PNI-associated biology, we correlated MYL4, CMYA5, and TNNT3 expression with the PNI score and related pathway modules(**Supplementary Figure S14**). At the single-cell level, super-enhancers were significantly activated in B cells, macrophages, NK cells, proliferating cells, endothelial cells, and fibroblasts (**Figure 4D**).

## 4 Discussion

This study integrates multi-omics data to systematically explore the regulatory mechanisms of PNI in HNSCC and its clinical significance.

In this study, the enrichment of pathways related to actin binding and cardiac muscle contraction was observed among the DEGs, suggesting that PNI may influence the mechanical basis of tumor migration or invasion by altering the calcium-dependent contraction-traction and cytoskeletal remodeling in tumor cells. Additionally, alterations in the neuroactive ligand-receptor interaction pathway suggest that these genes may regulate neuroimmune interactions, potentially affecting the infiltration and activity of immune cells within the tumor microenvironment. PNI might further promote tumor immune evasion by altering the expression of these neuroimmune genes, thereby accelerating tumor progression.

Through the integration of transcriptomic and proteomic analyses, we identified molecules such as MMP1, TFF3, and KRT24, which exhibited consistent changes at both omic levels. These molecules may play key roles in the PNI process and show potential as robust biomarkers for the diagnosis and treatment of PNI. Notably, MMP1 has been previously shown to be significantly overexpressed in pancreatic ductal adenocarcinoma compared to non-cancerous tissues, and MMP1 can significantly promote cell migration, invasion, and early PNI (12, 13). Recent work has shown that MMP1 is markedly upregulated in HNSCC and associated with poor survival, while fibroblast-derived MMP1 can actively enhance tumor-cell migration and invasion. Mechanistically, HNSCC-derived IL-8 was shown to activate STAT3 signaling in fibroblasts, thereby increasing MMP1 expression and promoting extracellular-matrix degradation and tumor invasion (14). These findings provide a plausible stromal framework for our observation that PNI-related tumors are enriched for extracellular-matrix remodeling programs. Beyond its pro-invasive role, emerging evidence also indicates that MMP1 may participate in immune escape in HNSCC. A recent mechanistic study demonstrated that SPHK1 upregulated MMP1 through ERK1/2 signaling, and that MMP1 contributed to sustained PD-L1 expression, reduced CD8+ T-cell-mediated killing, and poorer response to PD-1 blockade (15). Through LASSO regression analysis, we identified key proteins associated with survival, such as ADIPOQ, MB, PLIN1, and ADH1B. These proteins align with biomarkers reported in other studies, further validating the potential of PNI as a biomarker for predicting patient prognosis in clinical settings. For instance, ADIPOQ (adiponectin) and MB (myoglobin) have been reported to be closely related to patient survival in other cancers, suggesting that they may have similar roles in the context of PNI (16). In addition to its prognostic significance in HNSCC, ADIPOQ and other adipocyte-derived factors have been increasingly recognized for their roles in shaping the tumor immune microenvironment and influencing cancer progression, both through direct effects on tumor cells and via modulation of stromal and immune components within the microenvironment (17).

At the single-cell level, we observed significant remodeling of the tumor microenvironment (TME) before and after immunotherapy, particularly the shift in the ratio of fibroblasts to monocytes or macrophages, suggesting that PNI-associated genes may influence disease trajectory by reconstructing the cellular niche.It was reported that the coupling of cancer-associated fibroblasts (CAFs) with tumor neurogenesis or invasion, as well as the central role of monocytes or macrophages in PNI-related inflammation (18, 19). This study further demonstrated that, after risk stratification, enhanced MIF/CXCL signaling was observed in T cells from the low-risk group, suggesting that these cells are more active in chemotaxis and immune responses. In the high-risk group, macrophage-mediated interactions with multiple cell types were markedly reduced, suggesting a weakened immunoregulatory hub within the tumor microenvironment. Such disruption may compromise antigen presentation and/or chemokine-driven recruitment and activation of effector T cells, thereby contributing to attenuated immune signaling and impaired antitumor responses. Simulations of neural immune gene knockdown suggest that perturbations in these genes disrupt the “biogenesis or development” program, pointing to the upstream regulatory role of the neurogenesis-immune axis.

Spatial transcriptomics highlighted the enrichment of TLR2-related pathways at the tumor invasion front, suggesting that neuroimmune mechanisms are integral to PNI progression. Previous studies have shown that PNI outside the tumor is associated with worse prognosis, highlighting the importance of reporting PNI at boundary or front sites (20). In the risk model for the invasion front of oral squamous cell carcinoma, PNI was involved in stratification alongside the worst invasion pattern (WPOI), further reinforcing the clinical value of assessing the front areas (21).TLR2 has been shown to regulate tumor-associated inflammation and invasiveness (22). In the study, activation of TLR2 significantly enhanced the proliferation, migration, and invasion abilities of cancer cells, directly increasing tumor invasiveness (23). However, direct evidence linking TLR2 with the spatial localization of PNI remains relatively scarce. The observed enrichment of TLR2 at the invasion front in this study provides a more direct connection between the two. In the tumor microenvironment, particularly at the tumor invasive front, TLR2 may promote immune suppression, migration, and invasion of tumor cells through its interaction with TGFB1, suggesting that TLR2 may be upstream in the neuroimmune axis associated with PNI.

Recent reports have highlighted the role of neural-immune crosstalk in the PNI microenvironment (24). Moreover, TLR2 may act as an upstream mediator at the interface of inflammatory and neuroimmune signaling, potentially facilitating SE-linked transcriptional amplification of neuroimmune-related genes and thereby promoting coordinated remodeling at the invasion front. In this setting, TLR2-associated signaling may contribute to the formation of a PNI-related neuroimmune niche characterized by immune exclusion and stromal/neural reprogramming (25–28).

SEs are clusters of enhancers occupied by a large number of transcription factors and Mediator, which can significantly amplify the expression of key genes and reshape transcriptional programs associated with cell types and diseases (29).Previous studies have linked SEs to the reprogramming of tumor transcriptional programs, invasion or metastasis, and immune evasion (30–32). However, in the context of HNSCC, direct evidence linking the PNI phenotype with SE-driven 3D chromatin loops remains relatively insufficient. In this study, within an integrated multi-omics framework, we identified SE activity and SE-gene pairings through differential eRNA activity (tumor vs. normal) and Hi-C/TAD topological constraints. Notably, the PNI-associated SE-gene pair chr17:45390982-MYL4 was significantly correlated with overall survival. This suggests that SE targets and their associated gene networks could serve as potential intervention points for PNI risk stratification and therapy optimization, especially in conjunction with immunotherapy. Single-cell data further revealed that the cell-type-specific activation of SEs (in B cells, macrophages, NK cells, proliferating cells, endothelial cells, and fibroblasts) enhances the expression of target genes in these cell types via 3D chromatin architecture, suggesting that SEs play a role in the processes associated with PNI. Although direct evidence linking MYL4 to PNI remains limited, our findings raise the possibility that MYL4-associated SE programs may contribute to microenvironmental remodeling in HNSCC (33, 34). In particular, if MYL4-related regulatory activity is present in myofibroblast-like stromal cells or endothelial cells, it may be associated with changes in extracellular-matrix organization, stromal contractility, or vascular states that influence immune-cell trafficking (35–37). In this context, SE-mediated regulation of MYL4 could potentially help maintain stromal and vascular phenotypes that are less permissive to effective immune infiltration and more supportive of PNI progression. Consistent with prior evidence implicating myosin light chain family members in cell motility, tissue remodeling, and immune modulation, the MYL4-associated SE identified here may likewise be involved in shaping a PNI-related microenvironment characterized by enhanced invasiveness and immune suppression (38). This interpretation is further supported by the selective activation of this SE in macrophages, endothelial cells, and fibroblasts, which are recognized contributors to stromal organization and immune cell exclusion in solid tumors. Nevertheless, these interpretations should be considered hypothesis-generating and will require direct spatial and functional validation.

PNI is considered a significant pathological feature of poor prognosis and increased metastatic risk in HNSCC, and it suggests limited immune cell infiltration and enhanced immune evasion (39). Our data extend and reinforce this understanding at the population scale and through multi-omics integration: on one hand, PNI-positive patients exhibit reduced sensitivity to immunotherapy, and a significant association between PNI and the lack of response to dual immune therapy provides quantitative evidence for the clinical observation of “PNI-limited immunotherapy benefit.” On the other hand, the decreased levels of CD8^+^ T cells, B cells, and Tfh in the PNI-positive group align with the “immune cold tumor” phenotype described in the literature (40, 41).Recent studies have highlighted the progress and challenges of immunotherapy in HNSCC, emphasizing combination strategies to improve efficacy (28).Research indicates that effector T cells and inflammatory or pro-tumor macrophage characteristics are common in a more “active” immune microenvironment (42). The significant correlation we observed between CD8^+^ T cells and M0/M1 macrophages can be viewed as a spatial or sample-level co-occurrence of these immune components, consistent with the broader T-cell-myeloid axis collaboration described in the literature. Additionally, in both HNSCC and other cancers, higher levels of CD8^+^ Tumor-Infiltrating Lymphocytes are generally associated with better survival or a more active immune phenotype; macrophage polarization also correlates with prognosis and therapeutic efficacy (43–45). Therefore, the significant correlation between CD8^+^ T cells and M0/M1 macrophages may suggest that an immune-active phenotype is more commonly associated with better prognosis or potential sensitivity to immunotherapy at the population level.It is noteworthy that these immune infiltration patterns are highly consistent with the cell types involved in super-enhancer activation analysis, suggesting that enhancer-mediated transcriptional regulation may be a crucial mechanism underlying the immune microenvironment differences.In conclusion, CD8^+^ T cells may play a key role in the progression of PNI-associated tumors and immune therapy resistance through neuroimmune interactions. This finding not only uncovers new mechanisms of PNI in the immunological context of HNSCC but also provides potential directions for optimizing immunotherapy strategies.

This study not only provides a new perspective for a deeper understanding of the complex biological mechanisms underlying HNSCC, particularly the association between neuroimmune regulation and the formation of PNI, but also offers a theoretical basis for developing novel therapies targeting PNI and optimizing immunotherapy strategies. Future research will further explore the specific mechanisms by which neuroimmune genes regulate PNI through the TLR2 pathway, develop targeted drugs aimed at key node proteins or super-enhancer-target gene pairs, and translate these findings into clinical applications to provide more effective diagnostic and therapeutic options for HNSCC patients.

A limitation of this study is the relatively small sample size of the single-cell and spatial transcriptomic datasets, which may not capture the full biological heterogeneity of HNSCC. Moreover, some single-cell samples were obtained after nivolumab treatment, which could influence immune cell composition and intercellular signaling patterns. These factors should be considered when interpreting the findings, and future analyses using larger or independent cohorts will be necessary to validate the observed trends.

## 5 Conclusion

This study support PNI as a clinically relevant and biologically informative phenotype in HNSCC and provide a framework for improving prognostic stratification and guiding future mechanistic and therapeutic studies targeting neuroimmune and regulatory programs associated with PNI.

## Data Availability

The original contributions presented in the study are included in the article/**Supplementary Material**. Further inquiries can be directed to the corresponding author.
